# Association Between Fasting Insulin Levels and Handgrip Strength: A Cross-Sectional Study Using the Korean National Health and Nutrition Examination Survey

**DOI:** 10.3390/jcm14248653

**Published:** 2025-12-06

**Authors:** Hyang Rae Lee, Minjeong Ko, Seung-Kuy Cha, Taesic Lee

**Affiliations:** 1 Department of Family Practice and Community Health, Ajou University School of Medicine, Suwon 16499, Republic of Korea; hyangle1@ajou.ac.kr; 2 Department of Medicine, Yonsei University Wonju College of Medicine, Wonju 26426, Republic of Korea; kohmj0729@yonsei.ac.kr; 3 Department of Physiology, Yonsei University Wonju College of Medicine, Wonju 26426, Republic of Korea; 4 Department of Global Medical Science, Yonsei University Wonju College of Medicine, Wonju 26426, Republic of Korea; 5 Organelle Medicine Research Center, Yonsei University Wonju College of Medicine, Wonju 26426, Republic of Korea; 6 Division of Data Mining and Computational Biology, Department of Convergence Medicine, Yonsei University Wonju College of Medicine, Wonju 26426, Republic of Korea

**Keywords:** insulin resistance, sarcopenia, muscle quality, Mahalanobis, KNHANES

## Abstract

**Background**: Sarcopenia and metabolic dysfunction share common physiological mechanisms, and insulin resistance has been recognized as a major contributor to muscle loss. However, the independent association between circulating fasting insulin and muscle strength remains unclear. **Methods**: We analyzed data from 8343 Korean adults aged ≥ 20 years who participated in the 2015 and 2019 Korea National Health and Nutrition Examination Surveys. Multivariate outliers were removed using the Mahalanobis distance, and sampling weights were applied to account for the complex survey design. Multivariable linear regression models were constructed with progressive adjustments for demographic and metabolic covariates, and stratified analyses were conducted by age, BMI category, and diabetes status. **Results**: Crude models showed a weak positive association between fasting insulin and handgrip strength in both sexes. However, after adjustment for age and BMI, the association became significantly inverse and remained consistent in fully adjusted models. The inverse association was most pronounced in individuals aged ≤ 65 years, with BMI < 23 kg/m^2^, and without diabetes. **Conclusions**: Elevated fasting insulin levels were independently associated with lower handgrip strength in Korean adults. These findings suggest that hyperinsulinemia may reflect early metabolic changes linked to subclinical muscle weakness, warranting further longitudinal investigation.

## 1. Introduction

Skeletal muscle accounts for approximately 40% of total body weight and plays an essential role in movement, postural stability, and metabolic regulation, including glucose homeostasis and thermogenesis [1]. Sarcopenia, characterized by a progressive decline in skeletal muscle mass, strength, and function, is clinically defined as a loss of muscle quantity and quality that impairs physical performance, according to the EWGSOP and AWGS consensus criteria [2,3]. Its prevalence increases markedly with age and affects approximately 10–20% of older adults worldwide. Sarcopenia is now recognized as an independent clinical condition rather than an inevitable consequence of aging and is associated with an increased risk of falls, disability, hospitalization, cardiovascular disease, mortality, and poor prognosis in cancer and other chronic diseases [4,5].

Among the diverse pathophysiological contributors to sarcopenia, metabolic dysfunction, particularly insulin resistance, has emerged as a key mechanism underlying progressive muscle loss. At the molecular level, insulin exerts anabolic effects through the PI3K/Akt/mTOR signaling pathway, promoting muscle protein synthesis and inhibiting proteolysis [6,7]. Insulin resistance impairs skeletal muscle health by inhibiting protein synthesis, reducing glucose uptake, and disrupting mitochondrial function, ultimately decreasing muscle mass and strength [8]. Although this association has been explored, most previous studies have relied on indirect surrogate markers of insulin activity, such as fasting glucose levels or the homeostatic model assessment of insulin resistance (HOMA-IR), rather than directly measured fasting insulin levels [9,10]. HOMA-IR, calculated as fasting insulin (µU/mL) × fasting glucose (mg/dL)/405, incorporates both glucose and insulin levels; however, it provides only an indirect estimate of insulin resistance and may obscure the independent anabolic or catabolic effects of circulating insulin [11,12]. In contrast, fasting insulin directly reflects compensatory hyperinsulinemia caused by impaired muscle insulin action [13]. Elevated fasting insulin sensitively signals subclinical metabolic stress within skeletal muscle, including mitochondrial redox imbalance, dysregulated glucose–fatty acid flux with lipotoxic intermediary accumulation, and chronic low-grade inflammation [14,15]. These disturbances converge to blunt mTOR-dependent protein synthesis, activate catabolic pathways, and progressively degrade muscle quality prior to detectable hyperglycemia [16].

Given that insulin is a potent anabolic hormone that directly stimulates muscle protein synthesis and aids glucose uptake in muscle tissue, the fasting serum insulin concentration may serve as a more specific early biomarker of oxidative and inflammatory metabolic stress affecting muscle health [13,16].

Across Asian populations, including Koreans, insulin resistance frequently develops at a relatively lower degree of obesity and is accompanied by early compensatory hyperinsulinemia [17]. This occurs despite characteristically lower pancreatic insulin secretion capacity and β-cell secretory reserve, because Asian individuals exhibit increased hepatic and skeletal muscle insulin resistance, heightened susceptibility to ectopic fat accumulation, and a higher prevalence of the metabolically obese normal-weight (MONW) phenotype even within the normal BMI range [17,18]. In Korea, a substantial proportion of individuals with metabolic abnormalities are non-obese, and hyperinsulinemia plays a central role in this phenotype [19,20].

Handgrip strength (HGS) is a simple, reliable, and widely used surrogate measure of muscle function and is recommended in both research and clinical practice as a screening tool for sarcopenia owing to its substantial predictive value for adverse health outcomes [3,21]. Despite the potential relevance of fasting serum insulin levels as a marker of metabolic dysfunction, population-based studies directly evaluating its association with HGS are limited, particularly in Asian populations with different metabolic profiles.

Therefore, this study aimed to investigate the relationship between fasting serum insulin concentration and HGS in a nationally representative sample of Korean adults. We also examined whether this association varied according to sex, age, BMI, and diabetes status to identify potential effect modifiers and gain further insights into the early metabolic factors contributing to muscle weakness.

## 2. Methods

### 2.1. Study Population

This study used data from the Korea National Health and Nutrition Examination Survey (KNHANES), a nationally representative cross-sectional survey conducted annually by the Korea Disease Control and Prevention Agency (KDCA). We integrated data from the 2015 (n = 7380) and 2019 (n = 8110) KNHANES cycles, as these were the two most recent datasets that included both fasting insulin levels and HGS measurements. The KNHANES datasets were accessed in February 2025, and all analyses were conducted using the most updated publicly available version of the database. Adults aged ≥ 20 years were eligible for inclusion. Participants with missing information for any of the following variables were excluded: age, sex, body weight, BMI, waist circumference, systolic blood pressure, diastolic blood pressure, fasting insulin, fasting glucose, total cholesterol, HDL-C, triglycerides, AST, ALT, hypertension status, diabetes status, dyslipidemia status, smoking, alcohol consumption, or sampling weights. In addition, biologically implausible observations were excluded based on predefined criteria before the final analytic sample was obtained. After applying these exclusion steps, a total of 8343 participants were included in the final analyses. Because KNHANES is a nationally representative dataset, all eligible participants who met the inclusion criteria were analyzed, and no separate sample size calculation was required. All KNHANES participants provided written informed consent. This study was conducted in accordance with the principles of the Declaration of Helsinki. Institutional Review Board (IRB) approval was waived because the KNHANES datasets are publicly available and fully de-identified.

### 2.2. Clinical and Laboratory Variables

All clinical and laboratory data were obtained from KNHANES, which employs standardized protocols and trained personnel for data collection. Demographic variables included age and sex. Anthropometric measurements included BMI, waist circumference, and systolic and diastolic blood pressure, which were assessed by trained examiners following standardized procedures. Clinical information, including diabetes status, use of antihypertensive medications, and use of lipid-lowering drugs, was collected using a structured questionnaire; diabetes was defined as fasting plasma glucose ≥ 126 mg/dL, physician-diagnosed diabetes, use of antidiabetic medications, or HbA1c ≥ 6.5%. The primary outcome variable, HGS, was measured using a digital dynamometer (T.K.K. 5401; Takei Scientific Instruments Co., Ltd., Niigata, Japan). Participants performed the test in a standing position, with their arms at their sides and elbows fully extended. Each hand was tested three times, and the highest value from the dominant hand was used for analysis. All biochemical assays in KNHANES were performed at certified central laboratories using standardized protocols and external quality-control procedures. Fasting plasma glucose was measured using a UV assay based on the hexokinase method, and serum insulin concentrations were determined by electrochemiluminescence immunoassay (ECLIA). Lipid profiles—including total cholesterol, triglycerides, and high-density lipoprotein cholesterol (HDL-C)—were analyzed using enzymatic colorimetric assays on automated analyzers. Liver function markers (aspartate aminotransferase (AST), and alanine aminotransferase (ALT)) were measured from fasting blood samples.

### 2.3. Statistics

Continuous variables were compared across the serum insulin quartile groups using one-way analysis of variance (ANOVA), and linear trends were assessed by assigning the median value of each quartile as a continuous predictor. The chi-square test was used to evaluate the differences in categorical variables. Pearson’s correlation coefficients were calculated to assess the linear associations between continuous variables. To account for the complex survey design and ensure that the findings were representative of the general Korean population, sampling weights provided by the KDCA were applied to all statistical analyses [22]. Linear trend analyses (*p*-for-trend) were conducted by assigning the median value of each insulin quartile as a continuous variable and by modeling insulin deciles (Q1–Q10) as ordinal predictors in multivariable linear regression.

Outliers were identified and excluded using a two-step procedure. First, principal component analysis (PCA) was performed on 11 clinical variables to reduce dimensionality, and the Mahalanobis distance (χ^2^ > 97.5th percentile, df = 2; χ^2^ = 7.38) was applied to the first two principal components to detect multivariate outliers. Second, univariate filtering was applied by excluding individuals with serum insulin levels exceeding three standard deviations from the mean, thereby minimizing the influence of extreme values.

To examine the association between serum insulin levels and HGS, we performed multivariate linear regression analyses using a stepwise adjustment. Five models were constructed as follows: Model 1 was unadjusted; Model 2 was adjusted for age; Model 3 was adjusted for age and BMI; Model 4 included antihypertensive medication use, diabetes status, and lipid-lowering drug use; and Model 5 was further adjusted for systolic blood pressure, fasting glucose, total cholesterol, HDL-C, triglycerides (TG), AST, and ALT levels.

To evaluate potential dose–response relationships, serum insulin levels were categorized into deciles and incorporated as ordinal predictors. Normality of continuous variables and regression residuals was assessed by visual inspection of histograms and Q–Q plots, and homogeneity of variances was evaluated using Levene’s test. Multicollinearity was examined using variance inflation factors (VIF). All statistical tests were two-tailed, and a *p*-value of <0.05 was considered statistically significant. Data analyses were conducted using R software (version 4.3.1; R Foundation for Statistical Computing, Vienna, Austria).

## 3. Results

### 3.1. Sex-Specific Analysis of the Serum Insulin-HGS Association with Outlier Exclusion

The distribution patterns of serum insulin concentrations were similar in both sexes, approximating a slightly right-skewed Gaussian distribution (Figure S1). The distribution of HGS differed significantly between the sexes (Figure S2A), whereas no significant sex-based differences were observed in serum insulin levels (Figure S2B). Serum insulin was further classified into normal range and hyperinsulinemia using a threshold of 10 μU/mL. The relationship between dichotomized insulin distribution and grip strength differed by sex (Figure S3). Therefore, we used sex-specific dataset for subsequent analyses.

**Figure 1:**
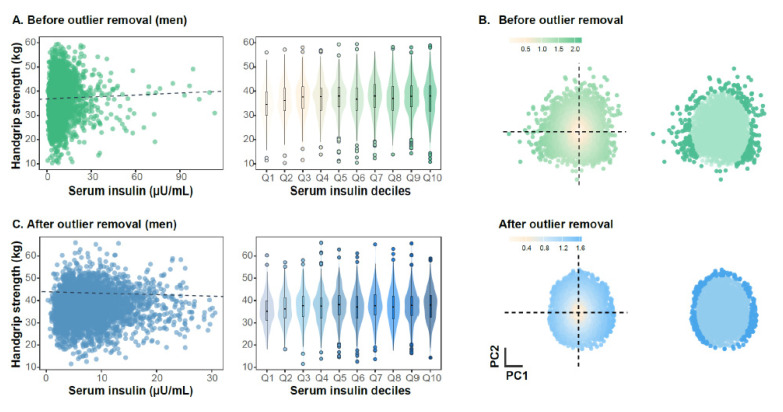
Male-specific association between serum insulin levels and handgrip strength and identification of multivariate outliers ((**A**–**C**) present unadjusted analyses). (**A**) Scatter plots indicating the distribution of serum insulin levels and handgrip strength among men. Each point denotes a male participant. The dashed line represents the Pearson correlation coefficient (PCC) of insulin and HGS. The x-axis of the boxplot displays the deciles of serum insulin. (**B**) Mahalanobis distance-based outlier removal process. Each point represents a male participant. The point color in the left panel is proportional to the Euclidean distance from the origin. The dense color points in the right panel indicate outliers determined by the Mahalanobis distance (χ^2^ > 97.5th percentile, df = 2). (**C**) Analysis of serum insulin levels and HGS in men after outlier removal.

**Figure 2:**
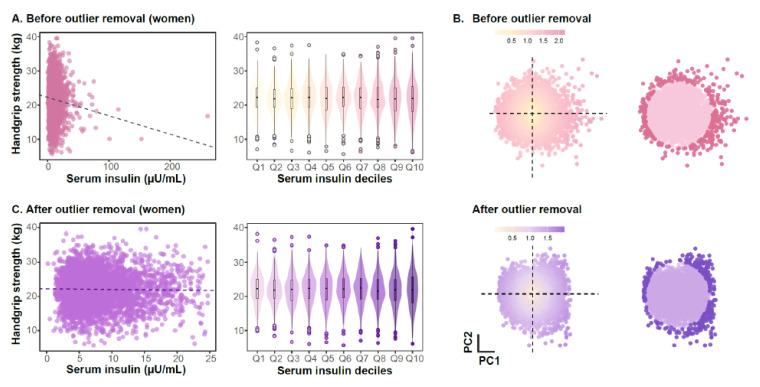
Female-specific association between serum insulin levels and handgrip strength and identification of multivariate outliers. ((**A**–**C**) present unadjusted analyses). (**A**) Scatter plots indicating the distributions of serum insulin levels and handgrip strength among women. Each point denotes a female participant. The dashed line represents a Pearson correlation coefficient (PCC) value of insulin and HGS. The x axis of boxplot displays the deciles of serum insulin. (**B**) Mahalanobis distance-based outlier removal process. Each point represents a female subject. The point color in the left panel is proportional to Euclidean distance from the origin. The dense color points in the right panel indicates outliers determined by Mahalanobis distance (χ^2^ > 97.5th percentile, df = 2). (**C**) Analysis of serum insulin levels and HGS in women after outlier removal.

In Korean men, serum insulin levels were not significantly correlated with HGS (Pearson’s r = 0.027, *p* = 0.091; Figure 1A). In contrast, insulin levels showed a negative associated with HGS in women (Pearson’s r = −0.054, *p* < 0.001, Figure 2A). Multiple variables, including BMI, systolic blood pressure, insulin, glucose, total cholesterol, HDL-C, TG, AST, and ALT, demonstrated right-skewed distributions, indicating the presence of outliers (Figure S4). Therefore, we eliminated outliers through a two-step procedure. First, we performed PCA to reduce the dimensionality of the 11 clinical variables into a two-dimensional space. The first two principal components explained approximately 39% of the total variance, indicating that the multivariate structure of the clinical variables could be effectively represented in a two-dimensional space for visualizing data patterns and identifying outliers. Second, we applied the Mahalanobis distance to the first two principal components, identifying 168 men and 190 women as outliers (Figure 1B and Figure 2B). Although multivariate outlier detection using the Mahalanobis distance removed many atypical observations, several extreme insulin values persisted (Figure S5). Because insulin displayed a right-skewed distribution and exerted disproportionate influence on the regression coefficients, we additionally excluded individuals with serum insulin levels exceeding three standard deviations from the mean to further minimize the influence of extreme values (Figure S6). The filtered dataset was then used for subsequent analyses of the association between serum insulin levels and HGS. In this refined sample, Pearson correlation and univariate linear regression revealed a positive association between insulin levels and HGS in men, whereas the association was negative in women (Pearson’s r = 0.047, *p*-value = 0.005, Figure 1C; Pearson’s r = −0.018, *p*-value = 0.227, Figure 2C).

To evaluate whether insulin levels correlated linearly with HGS, we transformed continuous insulin data into deciles. Although HGS exhibited a slight upward trend across insulin deciles in men, the association did not reach statistical significance (β = 0.094, *p*-for trend = 0.051, Figure 1C). In women, no significant linear trend was observed (β = −0.016, *p*-for trend = 0.137, Figure 2C). Given these preliminary patters, we next examined whether baseline clinical characteristics differed across serum insulin. In Korean men, higher insulin quartiles were associated with greater HGS, higher adiposity (BMI and waist circumference), elevated blood pressure, and more adverse metabolic profiles, including higher glucose, total cholesterol, TG, AST, and ALT levels, as well as a greater prevalence of diabetes, antihypertensive medication use, and lipid-lowering drug use. HDL-C levels and age decreased across increasing quartiles (Table 1). In women, most clinical and metabolic parameters exhibited association patterns similar to those observed in men, with the exception of HGS and age, which did not demonstrate the same quartile-dependent trends (Table 1).

### 3.2. Multivariate Analysis of the Relationship Between Serum Insulin and HGS in Korean Adults

In Korean men, the weak positive association between fasting insulin levels and HGS observed in the unadjusted model became negative after adjusting for age and BMI (Model 2, Table 2). This inverse relationship remained consistent across all fully adjusted models (Model 3–5) (Table 2). Similarly, in Korean women, the initial positive association became negative after adjusting for age and BMI, and this inverse association persisted in all subsequent models (Table 2).

**Figure 3:**
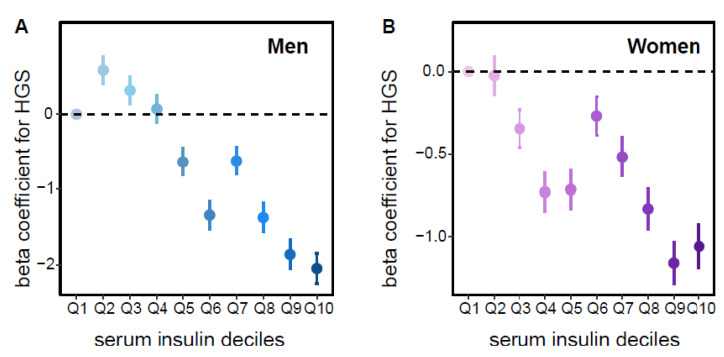
Association between serum insulin decile levels and handgrip strength. (**A**) Male participants. Each point represents the beta coefficient for the association between insulin (deciles of serum insulin levels on the x-axis of the risk plot) and HGS, calculated using multivariate linear regression. Insulin was modeled as the independent variable and HGS as the dependent variable, with covariates including age, BMI, AHM, DM, LLD, SBP, glucose, total cholesterol, HDL, TG, AST, and ALT. The upper and lower whiskers denote the confidence intervals estimated using the multivariate model. The plotted values represent fully adjusted regression coefficients rather than unadjusted descriptive means. (**B**) Female participants. Description is as in (**A**).

To further characterize the dose–response relationship between fasting insulin and HGS, we transformed continuous insulin levels into deciles and incorporated them into multivariate linear regression analysis. In Korean women, a clear inverse linear relationship was observed across insulin deciles after adjusting for medical and laboratory covariates (Figure 3B). In men, the association showed a non-linear pattern resembling an inverse J-shaped trend, with positive coefficients in the lower deciles and progressively stronger negative associations in the higher deciles (Figure 3A).

### 3.3. Stratified Analysis of the Insulin-HGS Relationship

**Figure 4:**
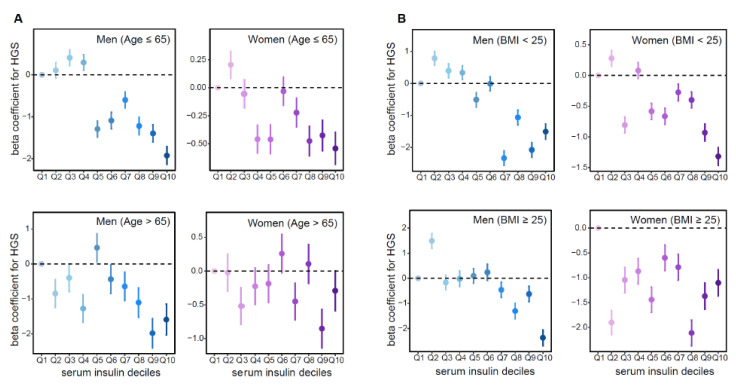
Stratified analyses of the association between serum insulin deciles and handgrip strength (HGS). Linear regression models were used to estimate the association between serum insulin deciles (Q1–Q10) and HGS. Serum insulin levels were categorized into deciles and included as independent variables in the analysis. Beta coefficients (dots) and 95% confidence intervals (whiskers) represent the estimated associations in each decile group. (**A**) Results stratified by sex and age. Upper left: Men ≤ 65 years; Upper right: Women ≤ 65 years; Lower left: Men > 65 years; Lower right: Women > 65 years. (**B**) Results stratified by sex and BMI. Upper left: Men with BMI < 25 kg/m^2^; Upper right: Women with BMI < 25 kg/m^2^; Lower left: Men with BMI ≥ 25 kg/m^2^; Lower right: Women with BMI ≥ 25 kg/m^2^.

To assess whether the association between fasting insulin and HGS was consistent across key demographic and metabolic subgroups, we performed stratified analyses according to age and BMI. In age-stratified analyses, an inverse association between serum insulin levels and HGS was observed in both younger and older men, and this trend was similarly maintained in women across all age groups (Table 3). The negative association across insulin deciles remained robust regardless of age group, demonstrating consistent inverse linear trends in both sexes (Figure 4A).

BMI-stratified analyses revealed that the inverse association between fasting insulin and HGS persisted in both sexes, regardless of obesity status, with a more substantial effect observed in the lower BMI group (Table 4). The dose–response trend across insulin deciles consistent within each BMI category (Figure 4B). To evaluate potential effect modification, interaction terms were added to the multivariate regression models. Insulin showed a negative association (β = −0.1416 in men; −0.0416 in women) with HGS, and the insulin × age (≤65 vs. >65 years) interaction was only significant in women (β = −0.034, *p*-value < 0.001), indicating that older age further strengthens this inverse insulin–HGS relationship. The interaction of insulin and BMI (≤25 vs. >25 kg/m^2^) was only significant in women (β = 0.083, *p* < 0.001), reflecting a weaker inverse association among individuals with higher BMI, consistent with the stronger negative slopes observed in the lower BMI group in Figure 4B.

**Figure 5:**
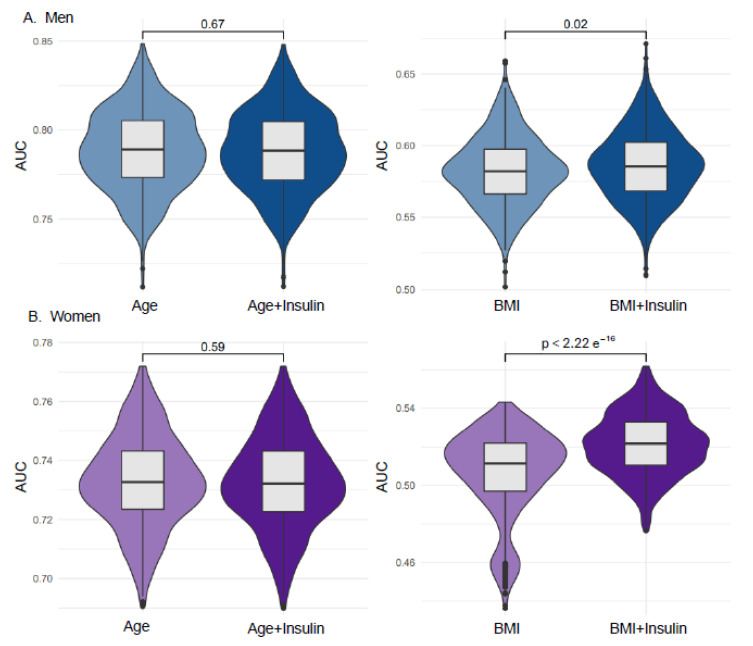
Predictive performance of serum insulin for low handgrip strength (HGS). Violin plots show the distribution of area under the ROC curve (AUC) values obtained from 1000 random 70:30 training-test splits. Higher AUC values on the y-axis indicate a better discrimination of low HGS values. The x-axis lists the covariates used for model construction with or without the inclusion of serum insulin. The numbers above the plots denote *p*-values comparing models that included serum insulin versus those that did not. (**A**) Results for men: left, Age vs. Age + Insulin; right, BMI vs. BMI + Insulin. (**B**) Results for women: left, Age vs. Age + Insulin; right, BMI vs. BMI + Insulin.

To determine whether adding serum insulin improves the prediction of low HGS status (<28 kg in men and <18 kg in women [2], we employed random sampling and logistic regression (LR) analysis using 1000 iterations of 7:3 train-test splits (Figure 5). Adding insulin to age resulted in only minimal and non-significant changes in AUC for both men and women (Figure 5). In contrast, incorporating serum insulin into BMI-based random sampling with LR yielded a significant improvement in predictive performance for low HGS, with the effect being more pronounced in women (Figure 5). These findings were further supported by DeLong’s test, which revealed no significant AUC difference in the age-only models (men: *p* = 0.155; women: *p* = 0.661). In contrast, when added to BMI, serum insulin consistently improved the predictive performance for low HGS in both sexes across the repeated LR analyses, with a greater enhancement in women. DeLong’s test confirmed these results, demonstrating significant AUC increases in BMI-based models for both men (ΔAUC = +0.035, *p* = 3.9 × 10^−12^) and women (ΔAUC = +0.036, *p* = 3.4 × 10^−6^).

## 4. Discussion

In this nationally representative sample of Korean adults, increased fasting serum insulin levels were significantly associated with reduced HGS. The inverse relationship was particularly evident among younger and non-obese individuals, and the trend became more pronounced after adjusting for age and BMI, suggesting that unadjusted models may underestimate the actual effect owing to confounding by age and body size. Given that BMI reduction is often accompanied by a loss of lean body mass, which may indirectly influence circulating insulin levels, these findings further highlight the complex interdependence between adiposity, muscle mass, and metabolic regulation [23]. Importantly, the overall associations remained consistent across stratified analyses based on sex, age and BMI status, which supports the robustness of our findings.

Sex-specific patterns were also observed in this study. In women, a negative linear relationship between insulin levels and HGS persisted, even after adjusting for multiple confounders. In men, the association was also significantly inverse, though the trend suggested a J-shaped pattern rather than a strictly linear relationship. This non-linear trend was statistically supported by the adjusted regression coefficients across insulin deciles. This result suggests that both low and high insulin levels in men may reflect metabolic impairments, such as β-cell dysfunction, chronic disease, or nutritional deficiencies [14,24]. Women may be less susceptible owing to the protective action of estrogen, differences in muscle fiber composition, and more stable metabolic adaptations [24,25].

These results are consistent with those of previous studies. For instance, Lazarus et al. first described an inverse association between fasting insulin levels and grip strength in the Normative Aging Study, with a relationship observed over a 23-year follow-up period [26]. A recent systematic review of 20 studies further confirmed that low HGS is consistently linked to metabolic syndrome and insulin resistance, primarily in older adults [27]. Although most existing studies have been conducted on middle-aged and older adults [28,29,30], similar associations have also been observed in younger populations. For example, in U.S. adolescents, lower grip strength is correlated with higher fasting insulin resistance and elevated blood glucose levels 2 h after a glucose tolerance test [31]. Collectively, these findings indicate that the insulin-muscle strength axis plays a role in the lifespan.

Multiple interconnected biological mechanisms may underlie the inverse association between fasting insulin levels and muscle strength. First, chronic hyperinsulinemia, a hallmark of early insulin resistance, desensitizes the phosphatidylinositol 3-kinase/AKT/mammalian target of rapamycin pathway in skeletal muscles, thereby impairing protein synthesis, muscle stem cell activation, and mitochondrial biogenesis [32,33]. This anabolic resistance decreases muscle regeneration and increases protein degradation, ultimately compromising muscle maintenance and function [33,34]. Second, insulin resistance disrupts cellular energy metabolism. Impaired glucose transporter type 4 translocation limits glucose uptake, whereas mitochondrial oxidative capacity decreases ATP production and increases oxidative stress [15,35]. Furthermore, suppression of peroxisome proliferator-activated receptor gamma coactivator-1α, a master regulator of mitochondrial biogenesis, impairs metabolic flexibility and muscular endurance [36]. Third, hormonal dysregulation undermines the anabolic environment. Insulin-like growth factor-1 (IGF-1) levels, which share downstream signaling cascades with insulin and promote muscle growth, are frequently reduced in conditions such as chronic diseases, inflammation, and malnutrition [11]. Despite sufficient insulin or IGF-1 levels, persistent hyperinsulinemia may cause receptor desensitization, thereby blunting anabolic signaling [37]. Finally, insulin resistance aggravates structural degeneration of muscle tissue through lipid accumulation and inflammation. Intramuscular fat deposition (myosteatosis), mediated by lipotoxic intermediates such as diacylglycerol and ceramides, disrupts muscle quality. These changes are compounded by chronic low-grade inflammation, which impairs muscle regeneration and promotes catabolic signaling [38,39,40]. Together, these mechanisms establish a vicious cycle of anabolic resistance, energy impairment, hormonal imbalance and inflammation-driven muscle loss. Importantly, the inverse association observed in non-obese and non-diabetic individuals suggests that reduced muscle strength may represent an early subclinical marker of insulin-related metabolic dysfunction, preceding overt diabetes or metabolic syndrome.

Notably, interaction analyses demonstrated that older age strengthened the inverse insulin–HGS association, while higher BMI attenuated it, especially among individuals with obesity (BMI > 25 kg/m^2^). The Bunkyo Health Study mainly included the elderly population, and successfully demonstrated the strong association between HGS and IR [41]. A systematic review and meta-analysis demonstrated that BMI and frailty exhibited a U-shaped relationship, and among individuals with a BMI below 25, further decreases in BMI were associated with an increased risk of frailty [42]. Given our findings, we suggest that in individuals with normal BMI, the phenotype of hyperinsulinemia may serve as an early predictive biomarker for a state of low muscle strength.

Importantly, this pattern of subgroup vulnerability was also reflected in the predictive performance analyses. When fasting insulin was added to age-only models, the changes in AUC were minimal and statistically non-significant, as confirmed by DeLong’s test. In contrast, incorporating fasting insulin into BMI-based models significantly improved the prediction of low HGS, with the effect being more pronounced in women. These results suggest that fasting insulin does not function as a universal biomarker across the entire population, but rather demonstrates meaningful discriminative value when metabolic load is taken into account [43,44]. Taken together, although the magnitude of the effect was modest, fasting insulin consistently enhanced risk stratification among individuals who are metabolically predisposed to declines in muscle strength, underscoring its potential utility for early screening before overt metabolic disease develops.

This study had several strengths. To enhance reliability and generalizability, we analyzed a large, nationally representative Korean cohort with standardized measurements of fasting serum insulin levels and HGS. Unlike previous studies that relied on surrogate indices, such as HOMA-IR, we directly measured fasting serum insulin levels, enabling a more accurate interpretation of its independent effects on muscle function. Additionally, HGS was assessed using a validated dynamometer, providing a clinically meaningful indicator of muscle performance and functional health status. Stratified analyses further identified subgroups, particularly younger, lean, and non-diabetic adults, in which insulin may serve as a sensitive marker of early metabolic dysfunction. Finally, by demonstrating these associations in individuals without obesity or diabetes, this study offers novel insights into subclinical metabolic processes during the pre-disease phase.

Nevertheless, the limitations of this study need to be addressed. First, the cross-sectional design precludes causal interference, rendering it uncertain whether hyperinsulinemia causes reduced muscle strength or vice versa. Accordingly, longitudinal studies are required to establish temporal causality. Second, direct measures of insulin resistance (e.g., clamp tests), muscle mass or composition, physical activity, and diet were unavailable, introducing the possibility of residual confounding factors. Finally, all variables were measured at a single time point, which may not have captured the long-term metabolic or functional trajectories.

In conclusion, we found that elevated fasting insulin levels were independently associated with reduced muscle strength in Korean adults. This inverse association was evident even among individuals without obesity or diabetes, suggesting that hyperinsulinemia and subtle declines in muscle strength may co-occur during the early stages of metabolic dysfunction. Evaluating fasting insulin levels may assist in identifying subclinical impairments in muscle function; however, the temporal relationship cannot be determined from cross-sectional data. Therefore, future longitudinal and interventional studies are required to clarify the directionality of these associations and to elucidate the underlying biological mechanisms linking insulin dysregulation and muscle strength.

## Figures and Tables

**Table 1:** Baseline characteristics by quartiles of serum insulin.

Variable	Men					Women				
	Insulin Quartiles				*p*-for Trend	Insulin Quartiles				*p*-for Trend
	Q1	Q2	Q3	Q4		Q1	Q2	Q3	Q4	
Handgrip strength (kg)	36.3 ± 0.2	37.3 ± 0.2	37.4 ± 0.3	37.5 ± 0.3	0.003	22 ± 0.1	22.1 ± 0.1	22.2 ± 0.1	21.9 ± 0.1	0.274
Age (years)	55 ± 0.5	51.6 ± 0.5	51.8 ± 0.6	50.1 ± 0.6	<0.001	51.5 ± 0.4	50.4 ± 0.5	51.8 ± 0.5	53.8 ± 0.5	<0.001
AHM, n (%)	177 (19.3%)	184 (20.1%)	236 (26.7%)	257 (28.7%)	<0.001	179 (14.7%)	199 (16.9%)	269 (23%)	371 (31.8%)	<0.001
Diabetes, n (%)	95 (10.4%)	87 (9.5%)	92 (10.4%)	100 (11.2%)	0.721	57 (4.7%)	64 (5.4%)	102 (8.7%)	122 (10.5%)	<0.001
LLD, n (%)	72 (7.9%)	87 (9.5%)	108 (12.2%)	112 (12.5%)	0.003	133 (10.9%)	132 (11.2%)	183 (15.7%)	232 (19.9%)	<0.001
BMI (kg/m^2^)	22.6 ± 0.1	23.7 ± 0.1	24.8 ± 0.1	26.6 ± 0.1	<0.001	21.6 ± 0.1	22.7 ± 0.1	23.6 ± 0.1	25.6 ± 0.1	<0.001
WC (cm)	82.1 ± 0.2	85.1 ± 0.2	88.6 ± 0.3	93.3 ± 0.3	<0.001	75.1 ± 0.2	78.2 ± 0.2	81 ± 0.3	86.3 ± 0.3	<0.001
SBP (mmHg)	120.2 ± 0.5	120.1 ± 0.5	122.2 ± 0.5	122.4 ± 0.5	<0.001	114.9 ± 0.5	115.8 ± 0.5	118.5 ± 0.5	121.2 ± 0.5	<0.001
Fasting glucose (mg/dL)	99.2 ± 0.8	102 ± 0.8	104.6 ± 0.8	106.6 ± 0.7	<0.001	92.2 ± 0.5	95.4 ± 0.5	99.4 ± 0.5	104.2 ± 0.6	<0.001
TC (mg/dL)	185.9 ± 1.1	188.9 ± 1.2	190.6 ± 1.2	192.2 ± 1.3	<0.001	193.5 ± 1	193.2 ± 1.1	194.3 ± 1.1	195.2 ± 1.1	0.181
HDL-C (mg/dL)	52 ± 0.4	48.2 ± 0.4	47.2 ± 0.4	44.3 ± 0.3	<0.001	59.4 ± 0.4	56.2 ± 0.4	53.9 ± 0.3	51 ± 0.3	<0.001
Triglycerides (mg/dL)	113.8 ± 2.5	149.3 ± 3.9	169 ± 4.5	189.6 ± 4.9	<0.001	88.5 ± 1.5	100.2 ± 1.6	116.6 ± 1.9	143.2 ± 2.4	<0.001
AST (U/L)	24.5 ± 0.4	23.7 ± 0.3	25.3 ± 0.4	28 ± 0.4	<0.001	21 ± 0.2	20.8 ± 0.2	21.1 ± 0.2	22.9 ± 0.3	<0.001
ALT (U/L)	20.5 ± 0.4	22.5 ± 0.4	26.3 ± 0.5	34.9 ± 0.7	<0.001	15.1 ± 0.2	16.1 ± 0.3	18 ± 0.4	21.5 ± 0.4	<0.001

General characteristics of men and women according to the insulin index quartiles. The participants were divided into four groups based on their serum insulin levels. For each sex, general characteristics are presented by serum insulin quartile, with continuous variables expressed as mean ± standard deviation and categorical variables as count (percentage), along with *p*-values. Continuous variables were compared using analysis of variance (ANOVA), and categorical variables were compared using the chi-square test. AHM, antihypertensive medication; LLD, lipid-lowering drug; BMI, body mass index; WC, waist circumference; SBP, systolic blood pressure; HDL-C, high-density lipoprotein cholesterol; AST, aspartate aminotransferase; ALT, alanine aminotransferase; TC, total cholesterol.

**Table 2:** Sex-specific multivariate linear regression analysis.

Models	Men		Women		Adjusted Variables
	Beta Coefficient	*p*-Value	Beta Coefficient	*p*-Value	
Crude	0.070	<0.001	0.000	0.404	-
Model 1	0.020	<0.001	0.020	<0.001	age
Model 2	−0.180	<0.001	−0.070	<0.001	Model 1 + BMI
Model 3	−0.177	<0.001	−0.062	<0.001	Model 2 + AHM, DM, LLD
Model 4	−0.177	<0.001	−0.063	<0.001	Model 3 + SBP
Model 5	−0.179	<0.001	−0.076	<0.001	Model 4 + Glc, TC, HDL-C, TG, AST, ALT

Multicollinearity among covariates was assessed using the variance inflation factor (VIF), and all VIF values were below 2.5, indicating no significant multicollinearity. Linear regression analysis was performed to separately evaluate the beta coefficient and *p*-value for the association between serum insulin levels and handgrip strength in men and women. Five linear models were used to examine the independent relationship between serum insulin levels and handgrip strength by incorporating different combinations of covariates. BMI, body mass index; AHM, antihypertensive medication; DM, diabetes mellitus; LLD, lipid-lowering drug; SBP, systolic blood pressure; Glc, fasting glucose; TC, total cholesterol; HDL-C, high-density lipoprotein cholesterol; TG, triglycerides; AST, aspartate aminotransferase; ALT, alanine aminotransferase.

**Table 3:** Linear trend between serum insulin deciles and handgrip strength stratified based on sex and age.

Model	Beta Coefficient	*p*-Value
Younger men	−0.23	<0.001
Older men	−0.15	<0.001
Younger women	−0.06	<0.001
Older women	−0.04	0.003

Serum insulin levels were divided into deciles (Q1–Q10) and treated as ordinal variables in the linear regression models to assess the linear trend, with handgrip strength (HGS) as the dependent variable. The analyses were stratified based on sex and age groups: younger men (≤65 years), older men (>65 years), younger women (≤65 years), and older women (>65 years). All models were adjusted for the same set of covariates. Beta coefficients represent the effect size per increase in insulin decile, and *p*-values indicate the statistical significance of the linear trend.

**Table 4:** Linear trend between serum insulin deciles and handgrip strength stratified based on sex and BMI.

Model	Beta Coefficient	*p*-Value
Men (non-obese)	−0.30	<0.001
Men (obese)	−0.26	<0.001
Women (non-obese)	−0.11	<0.001
Women (obese)	−0.06	<0.001

Serum insulin levels were divided into deciles (Q1–Q10) and treated as ordinal variables in the linear regression models to assess the linear trend, with handgrip strength (HGS) as the dependent variable. Analyses were stratified based on sex and age groups: non-obese (BMI ≤ 25 kg/m^2^) and obese (BMI > 25 kg/m^2^). All the models were adjusted for the same set of covariates. Beta coefficients represent the effect size per increase in the insulin decile, and *p*-values indicate the statistical significance of the linear trend. BMI, body mass index.

## Data Availability

The codes used in this study are available from the corresponding author upon request. The data presented in this study are available from the Korea National Health and Nutrition Examination Survey (KNHANES) database (https://knhanes.kdca.go.kr). Access to the data requires approval from the Korea Disease Control and Prevention Agency (KDCA).

## Supplementary Materials

The following supporting information can be downloaded at: https://www.mdpi.com/article/10.3390/jcm14248653/s1, Figure S1: Sex-specific distribution of Insulin Status. Figure S2: Sex-specific distribution of handgrip strength and serum insulin levels. Figure S3: Handgrip strength differs by insulin status in men and women: results from sex-stratified *t*-tests. Figure S4: Variable distribution before outlier exclusion. Figure S5: Variable distribution after outlier exclusion (Mahalanobis distance-based). Figure S6: Sex-specific insulin distribution before and after 3SD-based outlier removal. Table S1: Linear trend between serum insulin deciles and handgrip strength stratified by sex and age. Table S2: Linear trend between serum insulin deciles and handgrip strength stratified by sex and BMI.
