# Supplementary material for: Association Between Fasting Insulin Levels and Handgrip Strength: A Cross-Sectional Study Using the Korean National Health and Nutrition Examination Survey
Source: J Clin Med. 2025 Dec 6;14(24):8653. doi: 10.3390/jcm14248653 (PMC12734391; doi:10.3390/jcm14248653)
Supplement: Supplementary file 1 [file jcm-14-08653-s001.zip › jcm-3982107-supplementary.pdf]

Association Between Fasting Insulin Levels and Handgrip Strength: A Cross-Sectional Study Using the Korean National Health and Nutrition Examination Survey

Hyang Rae Lee 1,†, Minjeong Ko 2,†, Seung-Kuy Cha 3,4,5,* and Taesic Lee 5,6,*

1 Department of Family Practice and Community Health, Ajou University School of Medicine, Suwon 16499, Republic of Korea; hyangle1@ajou.ac.kr
2 Department of Medicine, Yonsei University Wonju College of Medicine, Wonju 26426, Republic of Korea; kohmj0729@yonsei.ac.kr
3 Department of Physiology, Yonsei University Wonju College of Medicine, Wonju 26426, Republic of Korea
4 Department of Global Medical Science, Yonsei University Wonju College of Medicine, Wonju 26426, Republic of Korea
5 Organelle Medicine Research Center, Yonsei University Wonju College of Medicine, Wonju 26426, Republic of Korea
6 Division of Data Mining and Computational Biology, Department of Convergence Medicine, Yonsei University Wonju College of Medicine, Wonju 26426, Republic of Korea
* Correspondence: skcha@yonsei.ac.kr (S.-K.C.); ddasic123@yonsei.ac.kr (T.L.); Tel.: +82-33-741-0295 (S.-K.C.); +82-33-741-1416 (T.L.)
† These authors contributed equally to this study

Index for the Supplementary Materials

Supplementary Figures 2

Supplementary Figure S1. Sex-specific distribution of Insulin Status 2

Supplementary Figure S2. Sex-specific distribution of handgrip strength and serum insulin levels. 3

Supplementary Figure S3. Handgrip strength differs by insulin status in men and women: results from sex-stratified t-tests. 4

Supplementary Figure S4. Variable distribution before outlier exclusion. 5

Supplementary Figure S5. Variable distribution after outlier exclusion (Mahalanobis distance-based) 6

Supplementary Figure S6. Sex-specific insulin distribution before and after 3SD-based outlier removal 7

Supplementary Tables 8

Supplementary Table S1. Linear trend between serum insulin deciles and handgrip strength stratified by sex and age

8 Supplementary Table S2. Linear trend between serum insulin deciles and handgrip strength stratified by sex and BMI 9

Supplementary Figures

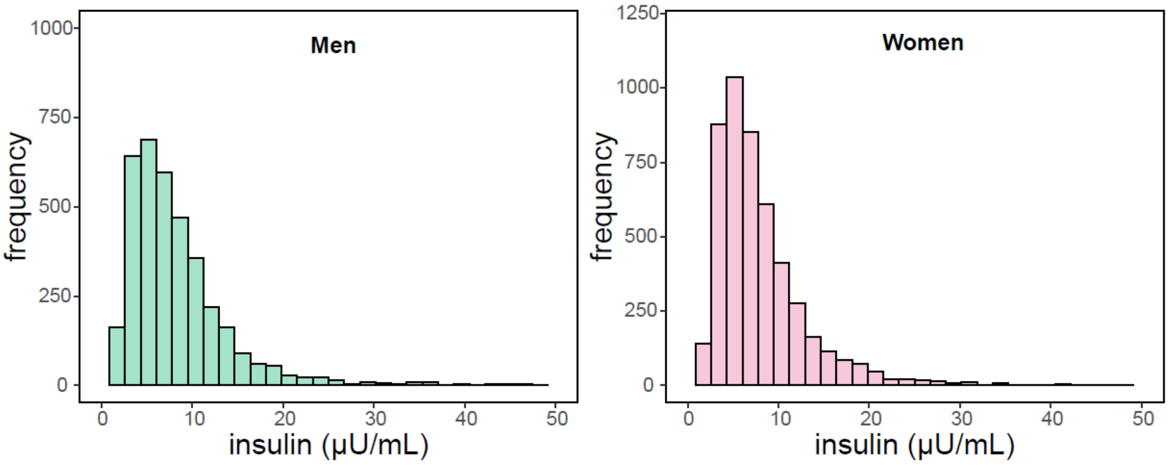

Supplementary Figure S1. Sex-specific distribution of Insulin Status. Distribution of serum insulin levels (μU/mL) in men and women.

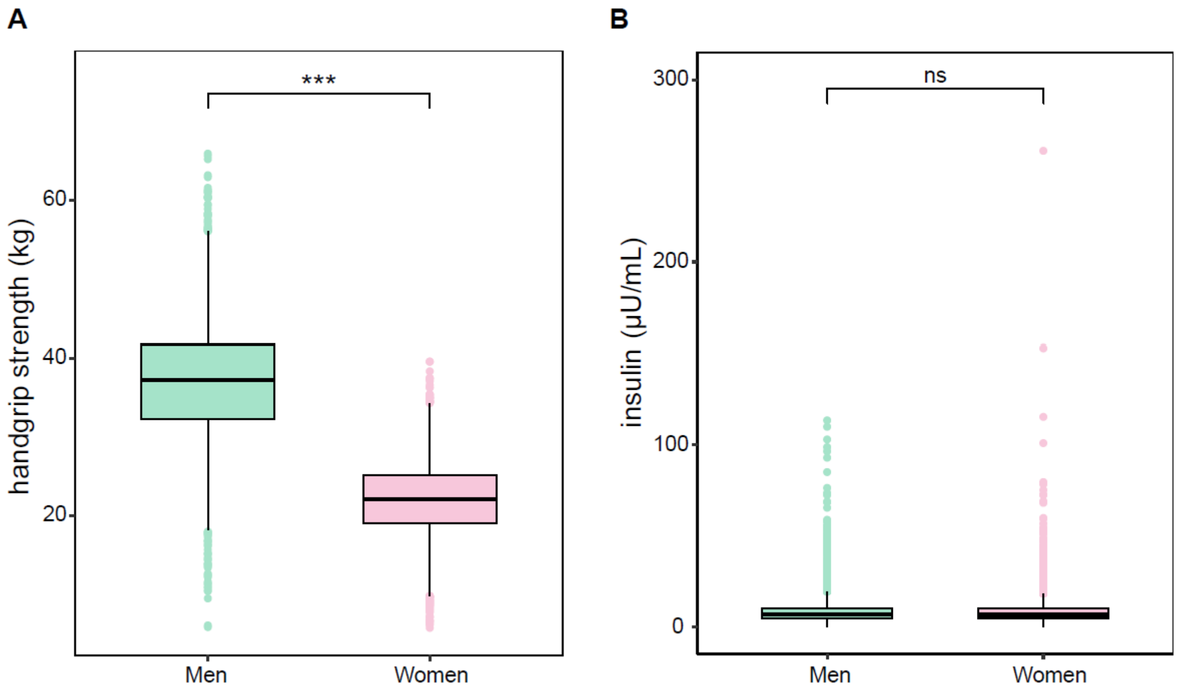

Supplementary Figure S2. Sex-specific distribution of handgrip strength and serum insulin levels.

Boxplots showing sex-specific distributions of handgrip strength (A) and serum insulin levels (B). Men exhibited significantly higher handgrip strength compared to women ($p < 0.001$, Student's t-test), as denoted by triple asterisks (***). No significant sex difference was observed in serum insulin levels ($p > 0.05$). Each dot represents an individual measurement, and outliers are defined as values exceeding 1.5× the interquartile range (IQR) beyond the upper or lower quartiles.

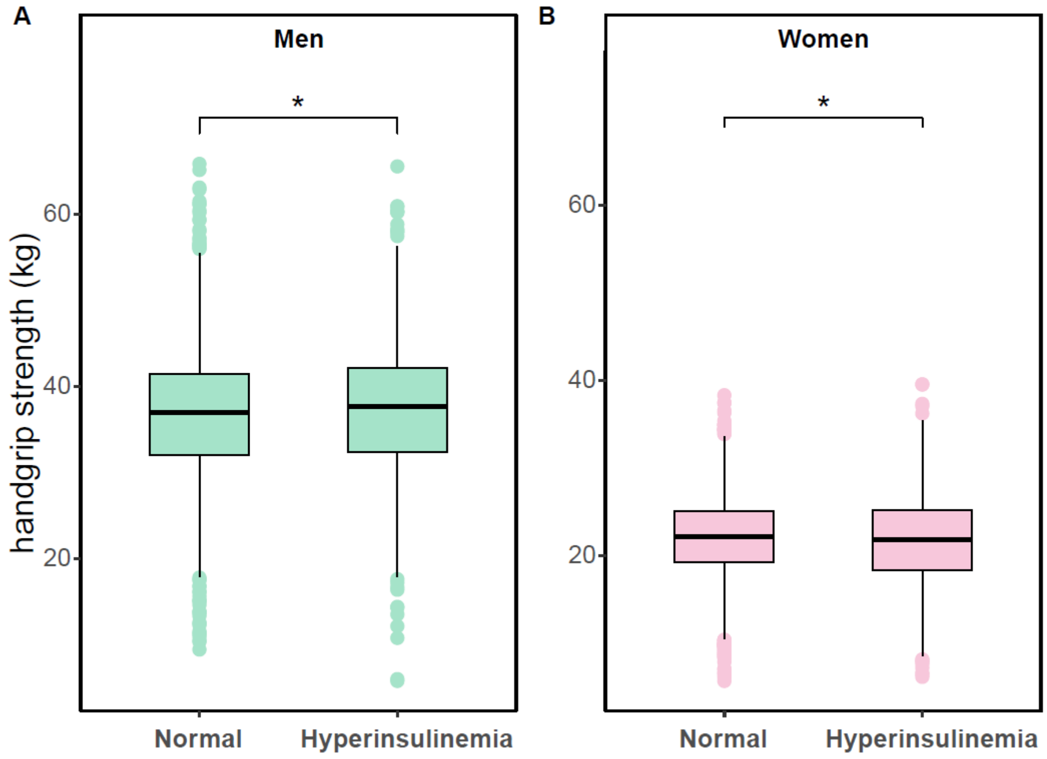

Supplementary Figure S3. Handgrip strength differs by insulin status in men and women: results from sex-stratified t-tests.

Serum insulin levels determined the grouping of participants into normal and hyperinsulinemia categories (cutoff: 10 μU/mL), and participants were stratified by sex. In men, the mean handgrip strength was 36.7 kg in the normal insulin group and 37.4 kg in the hyperinsulinemia group (*p*-value = 0.03). In women, the mean handgrip strength was 22.1 kg in the normal group and 21.7 kg in the hyperinsulinemia group (*p*-value = 0.05). Asterisk (*) indicates statistical significance at *p*-value≤0.0

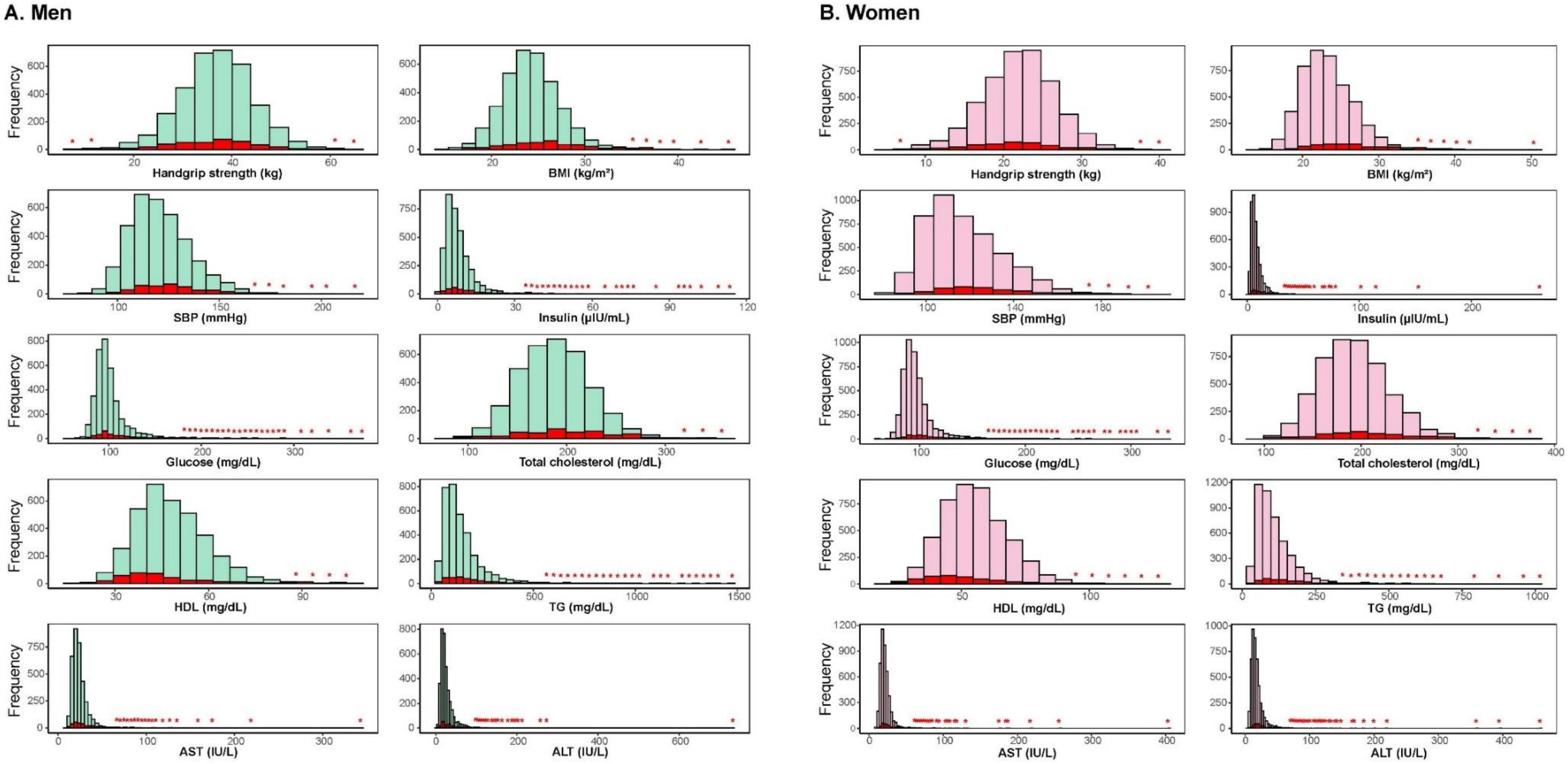

Supplementary Figure S4. Variable distribution before outlier exclusion.

Histograms showing the distribution of clinical variables prior to outlier exclusion, stratified by sex. Each plot represents the frequency distribution of a continuous variable, with potential multivariate outliers identified using Mahalanobis distance (based on all included variables). Outliers were defined as observations exceeding the 95th percentile of the chi-squared distribution with degrees of freedom equal to the number of variables. Red bars indicate values from participants classified as outliers, and red asterisks (*) denote instances where values exceeded 3 standard deviations.

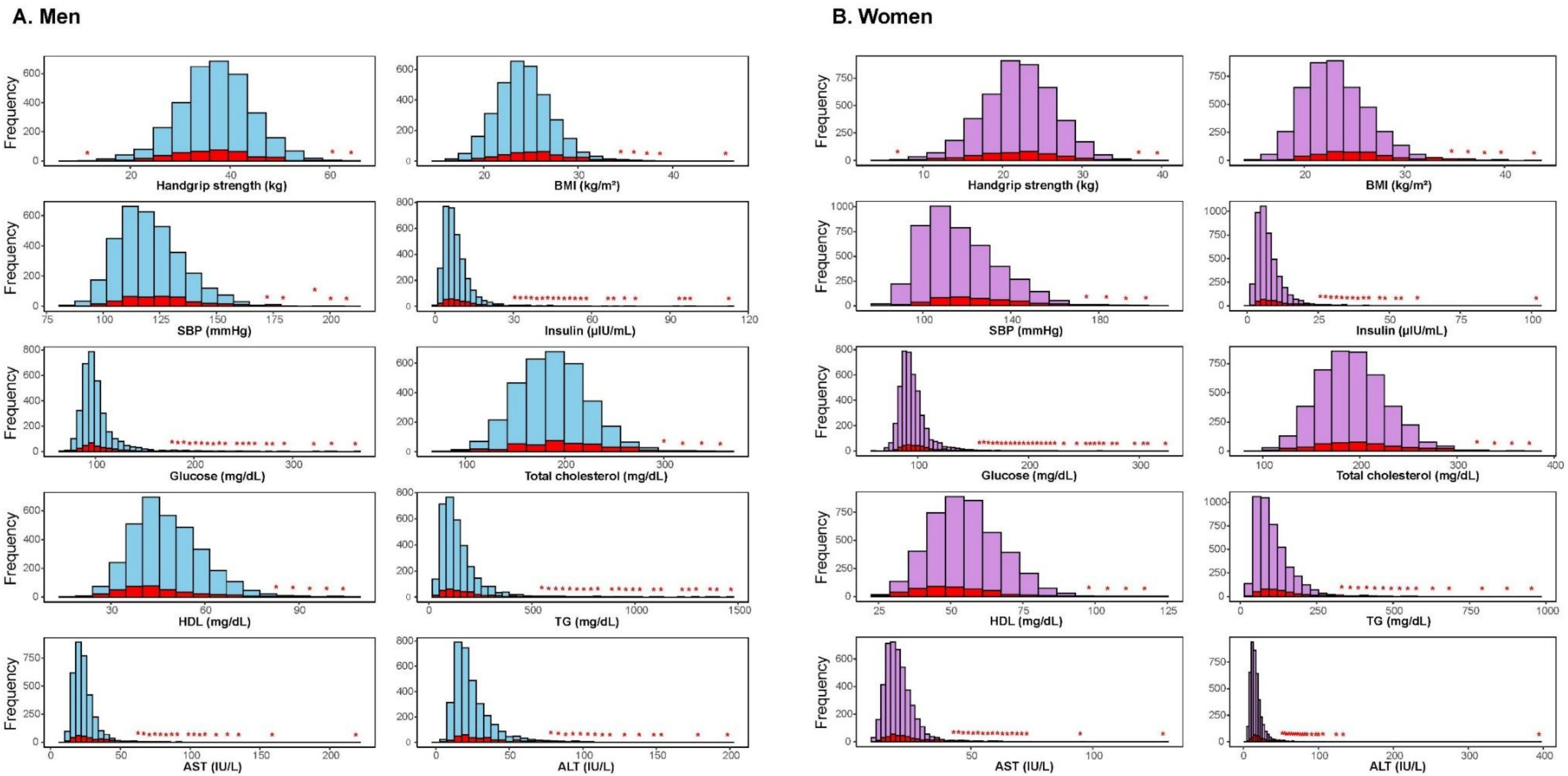

Supplementary Figure S5. Variable distribution after outlier exclusion (Mahalanobis distance-based)

Red bars denote secondary Mahalanobis outliers identified after excluding the first set.

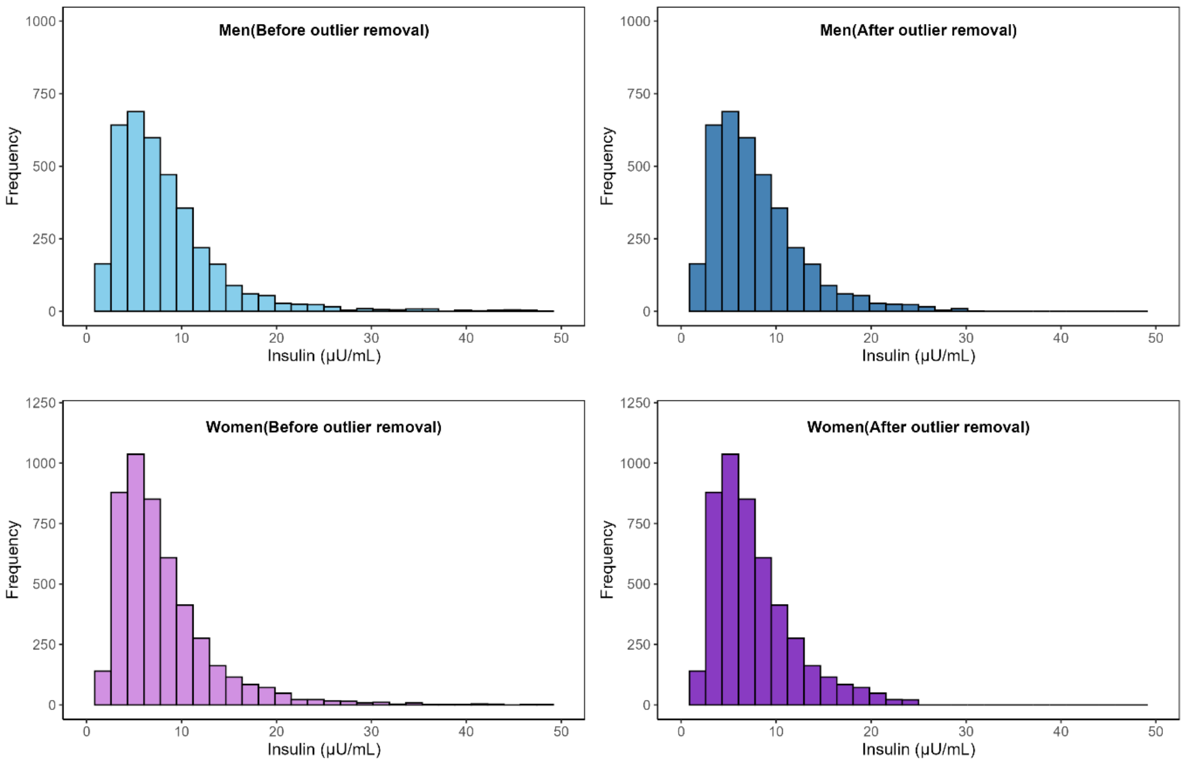

Supplementary Figure S6. Sex-specific insulin distribution before and after 3SD-based outlier removal

Histograms comparing the distribution of serum insulin levels (μU/mL) before and after outlier exclusion using the 3-standard-deviation (3SD) method. The top and bottom rows represent male and female participants, respectively. Outliers were defined as values exceeding the mean plus three times the standard deviation within each sex group. After exclusion, the right-side plots show a reduced upper tail, reflecting the removal of extreme insulin values.

Supplementary Tables

Supplementary Table S1. Linear trend between serum insulin deciles and handgrip strength stratified by sex and age

Model	Beta-coefficient	*p*-value
Young Men	-0.23	< 0.001
Old Men	-0.15	< 0.001
Young Women	-0.06	< 0.001
Old Women	-0.04	0.003

Serum insulin levels were divided into deciles (Q1–Q10) and treated as ordinal variables in linear regression models to assess the linear trend with handgrip strength (HGS) as the dependent variable. Analyses were stratified by sex and age groups: young men (≤65 years), old men (>65 years), young women (≤65 years), and old women (>65 years). All models were adjusted for the same set of covariates. Beta coefficients represent the effect size per increase in insulin decile, and p-values indicate the statistical significance of the linear trend.

Supplementary Table S2. Linear trend between serum insulin deciles and handgrip strength stratified by sex and BMI

Model	Beta-coefficient	*p*-value
Men (non-obese)	-0.30	< 0.001
Men (obese)	-0.26	< 0.001
Women (non-obese)	-0.11	< 0.001
Women (obese)	-0.06	< 0.001

Serum insulin levels were divided into deciles (Q1–Q10) and treated as ordinal variables in linear regression models to assess the linear trend with handgrip strength (HGS) as the dependent variable. Analyses were stratified by sex and age groups: non-obese (BMI ≤ 25), obese (BMI > 25). All models were adjusted for the same set of covariates. Beta coefficients represent the effect size per increase in insulin decile, and p-values indicate the statistical significance of the linear trend.
